# Study of Neuronal Apoptosis ceRNA Network in Hippocampal Sclerosis of Human Temporal Lobe Epilepsy by RNA-Seq

**DOI:** 10.3389/fnins.2021.770627

**Published:** 2021-11-12

**Authors:** Shengkun Yu, Yifei Gu, Tianyu Wang, Long Mu, Haiyang Wang, Shi Yan, Aoweng Wang, Jiabin Wang, Li Liu, Hong Shen, Meng Na, Zhiguo Lin

**Affiliations:** Department of Neurosurgery, The First Affiliated Hospital, Harbin Medical University, Harbin, China

**Keywords:** miRNAs, ceRNA, temporal lobe epilepsy, hippocampal sclerosis, lncRNAs

## Abstract

Hippocampal sclerosis (HS) is one of the most common pathological type of intractable temporal lobe epilepsy (TLE), often characterized by hippocampal atrophy, neuronal apoptosis, and gliogenesis. However, the molecular mechanisms of neuronal apoptosis in patients with HS are still not fully understood. We therefore conducted a pilot study focusing on the neuronal apoptosis ceRNA network in the sclerotic hippocampus of intractable TLE patients. In this research, RNA sequencing (RNA-seq) was utilized to quantify the expression levels of lncRNAs, miRNAs, and mRNAs in TLE patients with HS (HS-TLE) and without HS (non-HS-TLE), and reverse transcription-quantitative PCR (qRT-PCR). The interactions of differential expression (DE) lncRNAs-miRNAs or DEmiRNAs-mRNAs were integrated by StarBase v3.0, and visualized using Cytoscape. Subsequently, we annotate the functions of lncRNA-associated competitive endogenous RNA (ceRNA) network through analysis of their interactions with mRNAs. RNA-seq analyses showed 381 lncRNAs, 42 miRNAs, and 457 mRNAs were dysregulated expression in HS-TLE compared to non-HS-TLE. According to the ceRNA hypothesis, 5 HS-specific ceRNA network were constructed. Among them, the core ceRNA regulatory network involved in neuronal apoptosis was constituted by 10 DElncRNAs (*CDKN2B-AS1, MEG3, UBA6-AS1, etc.*), 7 DEmiRNAs (*hsa-miR-155-5p, hsa-miR-195-5p, hsa-miR-200c-3p, etc.*), and 3 DEmRNAs (*SCN2A, DYRK2, and MAPK8*), which belonging to apoptotic and epileptic terms. Our findings established the first ceRNA network of lncRNA-mediated neuronal apoptosis in HS-TLE based on transcriptome sequencing, which provide a new perspective on the disease pathogenesis and precise treatments of HS.

## Introduction

Temporal lobe epilepsy (TLE) is one of the most common and severe forms of drug-resistant focal epilepsies (Bell et al., 2011; Devinsky et al., 2016). Hippocampal sclerosis (HS) is the top prevalent neuropathological change found in patients with drug-resistant TLE, characterized by neuronal apoptosis, atrophy of hippocampi volume, and gliogenesis in the Cornu Ammoonis (CA)1, CA3, and CA4 (end folium) subfields of the hippocampus (Thom, 2004; Blümcke et al., 2013). Clinically, surgery remains an effective treatment option for approximately 60% of focal TLE with HS (HS-TLE), while 25∼30% of patients failed to achieve sustained seizure freedom after temporal lobe surgery (Engel et al., 2012; Yu et al., 2014; Dredla et al., 2016; Kang et al., 2016; Blumcke et al., 2017; Pruitt et al., 2017). Neuronal apoptosis is the most prominent pathological feature of HS-TLE and its exact molecular mechanism remains elusive. However, as compared to non-HS-TLE, patients with typical HS were more frequently associated memory impairment (Coras et al., 2014; Walker, 2015). Moreover, surgical resection or stereoelectroencephalography (SEEG) electrode implantations in epileptic hippocampus might unintentionally damage normal neurons and lead to vary degrees of memory dysfunction or cognitive impairment. Alternatively, many patients are ineligible for operation due to advanced age, coagulopathy or other contraindications. Continuing pharmacological therapies might cause significant adverse effects, such as myelosuppression, severe hepatotoxicity, and progressive cognitive impairment (Mehla et al., 2010). Consequently, there is an urgent need to better understand the exact molecular mechanisms underlying epileptogenesis and the neuronal apoptosis regulatory network in HS-TLE.

Recently, the advancement of high-throughput sequencing technologies have permitted transcriptome-wide analysis of comprehensive molecular features of HS-TLF. Nevertheless, prior study demonstrated that epileptogenesis frequently results from coding and non-coding RNA network abnormalities rather than single aberrant gene expression. Particularly, a subgroup of non-protein-coding transcripts with a size greater than 200 nucleotides (nt) could be classified into long non-coding RNAs (lncRNAs) (Wang et al., 2017), which have broad functional roles in numerous biological processes, including cell proliferation, metabolism and apoptosis, etc., MicroRNAs (miRNAs) are 18–22 nt endogenous non-coding RNAs, which bind to specific 3′-untranslated regions (UTRs) of target mRNAs, thereby modulating translation and/or mRNA decay. Currently there have been several sources of evidence showing that dysfunctions of lncRNAs and miRNAs might be associated with numerous neurological disorders, including cerebral infarction, glioma, and epilepsy (Jimenez-Mateos et al., 2012; Qi et al., 2017; Geng et al., 2018; Xu et al., 2018). For example, brain-specific miRNA-134 is up-regulated in human epileptic patients and silencing miRNA-134 relieves refractory seizures and pathological changes on hippocampus by inhibiting Limk1 translation. The hypothesis of competing endogenous RNA (ceRNA) introduced by Salmena et al. (2011) was presented as an emerging regulatory partners between non-coding RNA and coding RNA. According to ceRNA regulatory networks hypothesis, lncRNAs share complementary miRNA-binding sequences named miRNA-response elements (MREs) with mRNAs, by which pseudogenes crosstalk with protein-coding genes in various pathophysiological processes like epileptogenesis (Villa et al., 2019). Recently, Luo et al. (2019) reported a lncRNA-mediated ceRNA network in a rat TLE model contained 13 mRNAs, 10 miRNAs and 11 lncRNAs, which mainly involving the activity of voltage-gated potassium channels, and they suggested that lncRNAs may be a promising therapeutic target for TLE. Yet few studies direct focus on the ceRNA network of neuronal apoptosis in HS-TLE.

In the present study, we aimed to investigate ceRNA network in HS of TLE and apoptotic mechanisms of hippocampal neurons, for which we analyzed expression profiling (RNA-seq) data from 3 HS-TLE and 3 non-HS-TLE patients and integrated these results with our previous miRNA sequencing data. Through extensive bioinformatic analysis, we constructed an inferred lncRNA-miRNA-mRNA regulatory network. Intriguingly, all key molecules were significantly enriched for neuronal apoptosis-related pathways and epilepsy, which exhibited potentials as candidate markers that regulate apoptosis of hippocampal neurons and epileptogenesis in HS-TLE. Findings from this study may contribute to improved understanding of the intrinsic regulatory mechanisms underlying the pathogenesis of hippocampal neuron apoptosis, and provide potential targets for the treatment of HS-TLE, which require further experimental validation.

## Materials and Methods

**FIGURE 1:**
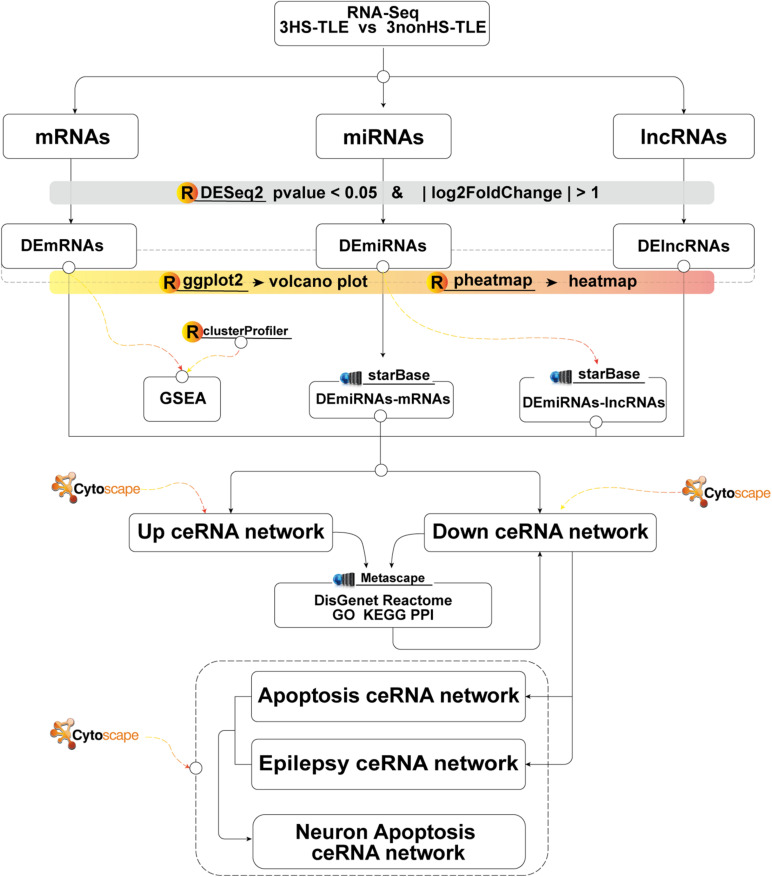
Flow chart of constructing lncRNAs-miRNAs-mRNAs ceRNA network.

In the following section, we describe the main steps in the construction of the ceRNA network for TLE. Firstly, we investigated the expression levels of lncRNAs, miRNAs, and mRNAs in human hippocampus samples of TLE using RNA-seq technique. Secondly, we constructed a ceRNA network through the integration of independently predicted interactions between DElncRNAs and DEmiRNAs and between DEmiRNAs and DEmRNAs based on the ceRNA hypothesis. Thirdly, we annotate the functions of lncRNA-associated ceRNA network through analysis of their interactions with mRNAs. Fourth, through the bioinformatic analysis of genes belonging to the neuronal apoptotic and epileptic cluster, the core neuronal apoptosis associated ceRNA network for TLE was constructed. The main analytical steps of our study were schematically reported in a technical roadmap (Figure 1).

### Clinical Information and Tissue Samples

During the period from 2014 to 2017, human hippocampus samples were obtained from patients with medically intractable TLE who underwent resection of presumed epileptogenic zone at our tertiary neurosurgical center following clinical seizure monitoring and systematic electrophysiological/imaging assessment as described in our previous study procedure (Tang et al., 2019). Briefly, the excised hippocampal specimen was divided into two parts along the long axis. A fraction of specimen was placed above nitrogen vapor for programmed cooling and eventually frozen in liquid nitrogen, the other part was fixed overnight in 4% paraformaldehyde at 4°C and then dehydrated and embedded in paraffin. Two neuropathologists independently reviewed each hippocampal specimen to examine whether HS and neuronal loss were present or not (immunohistochemical images were shown in Supplementary Figure 1). The body of the hippocampus was resected in each sample for next-generation sequencing and qRT-PCR. A total of 7 ILAE-1 and 7 no-HS samples were collected. We randomly chose 3 samples each type and prepared them for RNA-Seq. The Ethics Committee of the First Affiliated Hospital of Harbin Medical University approved this study, and all patients or their proxies signed an informed consent document.

### RNA Isolation and Quality Control

Total RNA was extracted from frozen hippocampus tissues using TRIzol reagent (Thermo Fisher Scientific, Waltham, MA, United States) following the manufacture’s protocol. The purity and quantity of RNA were measured by using a NanoPhotometer^®^ spectrophotometer (IMPLEN, CA, United States) and RNA integrity was evaluated by RNA Nano 6000 assay kit of the Bioanalyzer 2100 system (Agilent Technologies, CA, United States).

### Library Preparation for RNA and Small RNA Sequencing

A total of 5 μg RNA were utilized for each RNA sample. Firstly, ribosomal RNA (rRNA) was removed by Epicentre Ribo-zero^TM^ rRNA Removal Kit (Epicentre, United States). Subsequently, rRNA-depleted samples were converted to RNA-seq libraries using the NEBNext^®^ Ultra^TM^ Directional RNA Library Prep Kit for Illumina (NEB, United States) following the manufacturer’s recommendations. For small RNA libraries, a total amount of 3 μg RNA per sample was used as input material according to instructions from the NEBNext^®^ Multiplex Small RNA Library Prep Set for Illumina^®^ (NEB, Ipswich, MA, United States) as illustrated in our previous study (Tang et al., 2019). The clustering of the index-coded samples was performed on a cBot Cluster Generation System using TruSeq PE Cluster Kit v3-cBot-HS (Illumia). After cluster generation, the RNA libraries were sequenced on an Illumina Hiseq 4000 platform to 150 bp paired-end reads, and small RNA libraries were sequenced on an Illumina Hiseq 2500/2000 platform for paired end 50 bp reads.

### Data Processing

#### Quality Control (RNA and Small RNA)

For quality control, FASTQ raw reads were firstly processed through custom Perl and Python scripts. Clean data was obtained by removing adapter sequences, reads containing ploy-N and reads with low-quality from raw data. Simultaneously, quality of data was checked using FastQC. High quality clean data was used for downstream analysis.

#### Reads Mapping to the Reference Genome, Transcriptome Assembly and Quantification

RNA-seq reads were aligned to GRCh38.p13 human reference genome using Bowtie2 (Langmead and Salzberg, 2012) v2.4.2. We applied StringTie (v 2.1.5) (Kovaka et al., 2019) to assemble transcripts for the RNA mapped reads of each sample in reference-based mode. Transcript abundance was estimated using Gffcompare (Pertea and Pertea, 2020) v0.11.2.

Subsequently, small RNA reads were mapped to the reference sequence according to miRbase22 of human miRNAs using bowtie2 v2.4.2 (Langmead and Salzberg, 2012) with the default setting parameters. The counted reads were mapped to GENCODE genes and then calculated using the FeatureCount (Liao et al., 2014) programs.

### Bioinformatics Analysis

#### Screening for Differentially Expressed lncRNAs, miRNAs and mRNAs

Differential gene expression (DEG) analysis of raw read-counts from RNA-seq (lncRNA, miRNA and mRNA) data were performed using R packages DESeq2 (Love et al., 2014) with Wald significance tests. Benjamini-Hochberg correction was used to control the false discovery rate. Significant DEGs were determined if the results showed | log2FoldChange| > 1 and *P* value < 0.05. Volcano plots and heatmap of results were plotted using R package ggplot2 and pheatmap separatively.

#### Gene Set Enrichment Analysis for mRNAs

To initially understand the function of mRNAs, the differential analysis results of all mRNAs were sorted according to the range of Log2FoldChange, and performed hypergeometric analysis tests by clusterProfiler R package for Gene Set Enrichment Analysis (GSEA). Gene sets with an adjusted *p*-value < 0.05 were considered significant.

#### DEmiRNAs Targeting lncRNAs and mRNAs Analysis

Prediction of microRNA (miRNA) targets were conducted using starBase (Li et al., 2014). To our knowledge, starBase^1^ is one of the most comprehensive and widely used databases of miRNA-lncRNA and miRNA-mRNA interactions, with at least three supporting CLIP-Seq data sets, and this database integrates seven well-known miRNA targeting prediction programs: PITA, RNA22, miRmap, DIANA-microT, miRanda, PicTar and TargetScan. For each ceRNA pair, the hypergeometric test was applied utilizing (Sumazin et al., 2011) with the *P*-value determined by the following formula, which contains four parameters:(i) N is the total number of miRNAs utilized to predict targets; (ii) K is the number of miRNAs that interact with given mRNAs; (iii) n is the number of miRNAs that interact with the candidate ceRNA of the chosen gene; (iv) c is the common miRNA number shared between these two genes. Each target gene should be retrieved in at least 3 independent databases. All genes were corrected for the false discovery rate (FDR), and only FDR-adjusted *P*-values < 0.05 were considered statistically significant.

p=∑i=cm⁢i⁢n⁢(K,n)()iK()n-iN-K()nN

#### Establishment of the ceRNAs Network

According to ceRNA hypothesis, the downregulated ceRNA sub-network consisted of down-regulated DElncRNAs, up-regulated DEmiRNAs and down-regulated DEmRNAs. Whereas upregulated ceRNA sub-network was comprised of up-regulated DElncRNAs, down-regulated DEmiRNAs, and up-regulated DEmRNAs. The ceRNA network was visualized with Cytoscape software (Shannon et al., 2003).

#### Protein-Protein Interaction, Functional Enrichment, and Gene Modules Analysis

To annotate the function of the up and down-regulated ceRNA sub-networks mediated by DElncRNA, Metascape database^2^ was used for Gene ontology (GO) and Kyoto Encyclopedia of Genes and Genomes (KEGG) pathway, protein-protein interaction (PPI), and disease enrichment analysis The parameters were set as default. Briefly, *p*-values were calculated using the accumulative hypergeometric distribution, and Multiple testing adjustments (*q*-value) were calculated using the Banjamini-Hochberg method (Hochberg and Benjamini, 1990). The top 20 ranked GO terms were selected according to *p*-value. PPI enrichment analysis was performed with the following databases: STRING (Szklarczyk et al., 2019), BioGrid (Stark et al., 2006), OmniPath (Li et al., 2017), and visualized using Cytoscape software (Shannon et al., 2003). To identify densely connected sub-networks, the Molecular Complex Detection (MCODE) method (Bader and Hogue, 2003) algorithm was applied. An enrichment analysis of pathways and processes has been performed on each MCODE component individually.

#### Establishment of Neuron Apoptotic Core ceRNA Network

All differential genes belonging to apoptosis cluster obtained from GO term were selected. According these genes were also in the downregulated ceRNA sub-network to construct the neuronal apoptosis associated ceRNA network. Likewise, the epilepsy associated ceRNA network was constructed through selecting for differentially expressed genes belonging to epileptic cluster collected from DisGeNET and which were all in the downregulated ceRNA sub-network. Following this, epilepsy and apoptosis associated ceRNA networks were integrated according to shared nodes to form a core ceRNA network related to neuronal apoptosis in HS-TLE. The Cystoscope (version 3.8.2) software was used to visualize these ceRNA sub-networks.

#### Quantitative Real-Time Reverse-Transcription Polymerase Chain Reaction Validation

The top three differentially expressed RNAs from each category in the neuronal apoptosis core ceRNA network were selected for qRT-PCR validation according to the absolute value of Log2FoldChange, while genes that have been verified in our previous studies or defined as *de novo* non-coding RNAs were excluded from further validations.

Total RNAs were extracted from 6HS and 6no-HS patients with EZNA^TM^ Total RNA kit II (Omega Bio-Tek). Subsequently mRNAs and lncRNAs were reverse transcribed into single-stranded cDNA following the operating instructions of the Reverse Transcription Kit (Toyobo Life Science, Japan). RNase free water was run in parallel as negative control, and GAPDH was applied as an inner control for mRNA and lncRNA separately. Then qRT-PCR were performed using SYBR Green Real-Time PCR Master Mix (Toyobo Life Science, Japan) with at least three independent replicates for each reaction. The real-time PCR cycling conditions were as follows: 95°C for 10 min, 40 cycles at 95°C for 15 s, 60°C for 1 min. The melt curve stage was set as follows: 95°C for 15 s, 60°C for 60 s, and 95°C for 15 s. The relative quantities of each RNA in comparison to GAPDH were calculated by equation 2^–ΔΔ*CT*^.

MiRNA from all samples were firstly reverse transcribed using with Poly(A) Polymerase by miDETECT A TrackTM qRT-PCR Starter Kit (Ribobio Guangzhou, People’s Republic of China) as recommended by the manufacturer. MicroRNA-specific primers (Guangzhou RiboBio, People’s Republic of China) were used for reverse transcription of selected miRNA. Then qRT-PCR was performed with SYBR Green Real-Time PCR Master Mix (Toyobo Life Science, Japan) with at least three independent replicates for each reaction. The endogenous, small-nuclear RNA U6 was used for the normalization of all miRNAs. The real-time PCR cycling conditions were as follows: 95°C for 10 min, 40 cycles at 95°C for 2 s, and 60°C for 20 s, 70°C for 10 s. The melt curve stage was set as follows: 95°C for 15 s, 60°C for 60 s, and 95°C for 15 s. The relative fold change in the expression of miRNAs was determined using the 2^–ΔΔ*Ct*^ method.

The difference in lncRNAs, miRNAs and mRNAs expression between the HS-TLE group and the non-HS-TLE group was evaluated by GraphPad Prism 9.0 software. A value of *p* < 0.05 was considered statistically significant. The sequences of primers were listed in Supplementary Table 3.

#### Statistical Analysis

Most of the statistical analyses were carried out using the bioinformatic tools mentioned above, while the rest of the statistical analyses were performed by using R software (v 4.10) with default parameters in packages, such as DESeq2, clusterProfiler, etc., Identification of differentially expressed genes was assessed by DESeq2 package with Wald significance tests, and Benjamini-Hochberg FDR method was applied to adjust the *P*-value. Statistical analysis of qRT-PCR results were performed by two-tailed Student’s *t* test. Results were considered to be statistically significant when *p*-value was <0.05.

### Data Availability

The RNA raw sequencing data has been stored in the Sequence Read Archive (SRA) database with the registration number PRJNA699348 and raw sequence data of the miRNA has been submitted to GEO (GSE124507).

## Results

### Clinical and Pathological Characteristics

The clinical data of participants have already been described in our previous study (Tang et al., 2019). Briefly, human brain samples of 6 TLE patients (2 Female and 4 Men) drawn at random were utilized to investigated genome-wide transcriptomic characteristics by RNA-seq. The mean age of patients at operation were 28.33 ± 2.50 years, ranging from 24 to 31 years. We found no significant baseline difference between HS-TLE and non-HS-TLE group. Whereas, the average age at the first seizure of epilepsy was 7.33 ± 2.89 years in non-HS-TLE group and 26.00 ± 2.08 years in HS-TLE group with ranging from 9 to 29 years, and the difference between groups reached statistical significance (*p* < 0.05, Mann-Whitney test), which was consistent with previous studies (Blumcke et al., 2017). 83% patients were not successfully attained seizure control with triple antiepileptic drugs. Four patients received respective surgery for left anterior hippocampus, while the other two patients underwent right hippocampal resection. As described in our previous work, pyramidal neuronal density in hippocampal CA1 and CA4 regions significantly reduced in the HS group in comparison to no-HS group by measuring the relative optical density of immunohistochemical staining with *t*-test *p*-value < 0.001. The detailed clinical information and immunohistochemical results were shown in Supplementary Table 1 and Figure 1.

### Identification of Differentially Expressed lncRNA, miRNA, and mRNA

**FIGURE 2:**
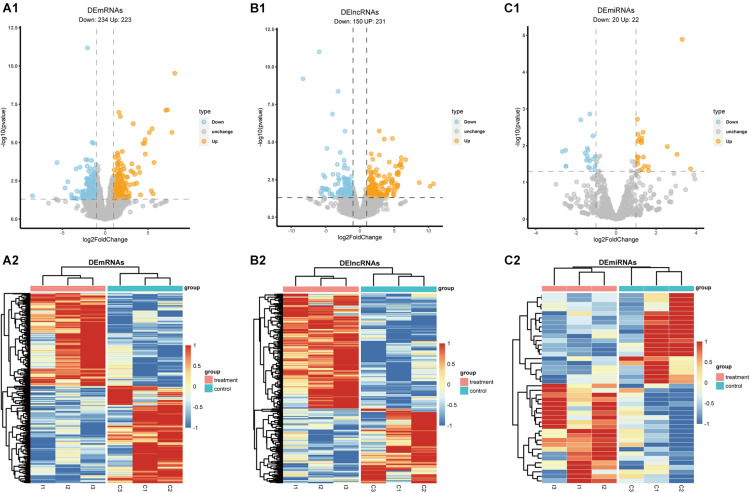
The volcano plot and heatmap of DEmRNAs, DElncRNAs, and DEmiRNAs. **(A1,B1,C1)** Volcano plot of DEmRNAs, DElncRNAs, and DEmiRNAs; **(A2,B2,C2)** heatmap of DEmRNAs, DElncRNAs, and DEmiRNAs.

To identify DElncRNAs, DEmiRNAs and DEmRNAs between HS and non-HS in TLE patients, we performed differential expression analysis by DEseq2 R package (Love et al., 2014). Our method identified 457 DEmRNAs (223 up-regulated and 234 down-regulated; Figure 2A1), 381 DElncRNAs (231 up-regulated and 150 down-regulated, Figure 2B1), and 42 DEmiRNAs (22 up-regulated and 20 down-regulated; Figure 2C1) across the transcriptomes of these two groups based on the criteria of | log2FoldChange| > 1 and *p*-value < 0.05. The volcano plot was utilized to visualize differential expressed gene and heatmap analysis showed significance levels of difference in global gene expression between HS versus non-HS groups (Figures 2A1–C2).

### Pathway Enrichment Analysis From GSEA

**FIGURE 3:**
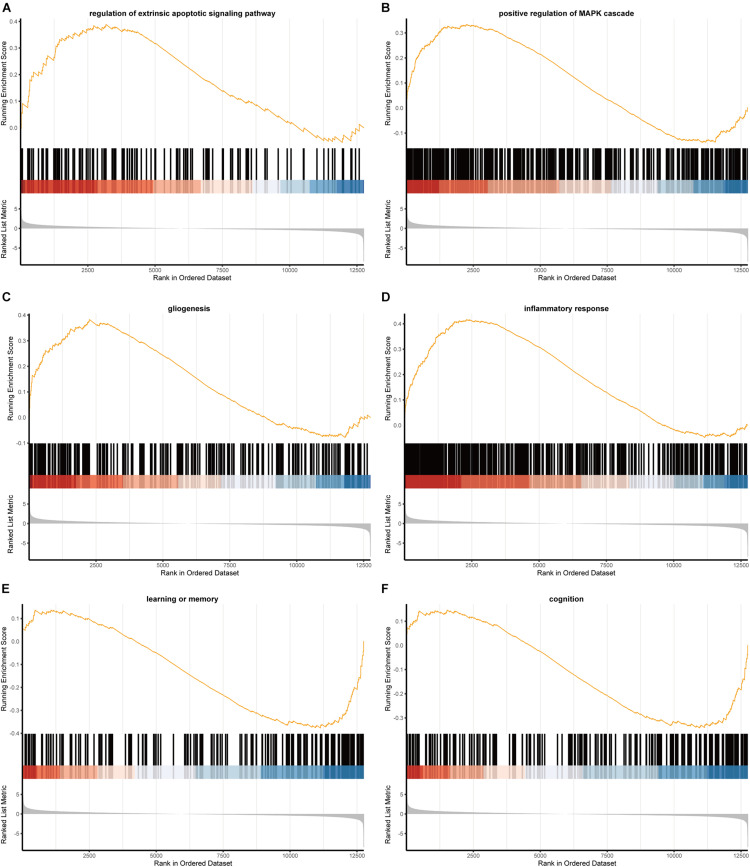
The GSEA results of mRNAs. **(A)** Regulation of extrinsic apoptotic signaling pathway; **(B)** positive regulation of MAPK cascade; **(C)** gliogenesis; **(D)** inflammatory response; **(E)** learning or memory; **(F)** cognition.

The results of GSEA pathway enrichment analyses were shown in Figure 3, which displayed 324 GO terms and 38 canonical pathways. The differentially expressed RNAs were significantly enriched in pathways related to epilepsy and HS, which included extrinsic apoptotic signaling pathway, positive regulation of MAPK cascade, gliogenesis, inflammatory response, learning or memory, and cognitive functions.

### Construction of DElncRNAs-DEmiRNAs-DEmRNAs ceRNA Regulatory Network

**FIGURE 4:**
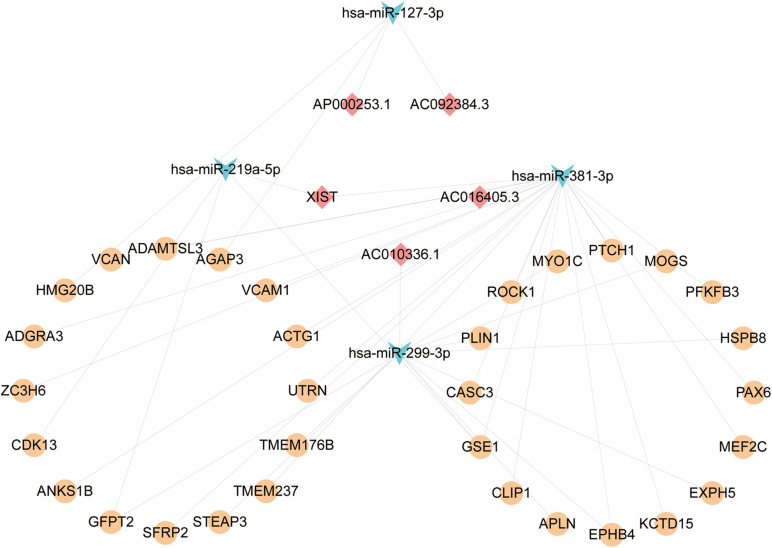
The Up lncRNA-miRNA-mRNA ceRNA network. Squares represent DElncRNAs, whereas triangles represent DEmiRNAs, and circles represent DEmRNAs. Light blue indicates down-regulated DEmiRNAs. Light red represents Down-regulated DElncRNAs. Orange-yellow indicates up-regulated DEmRNAs.

**FIGURE 5:**
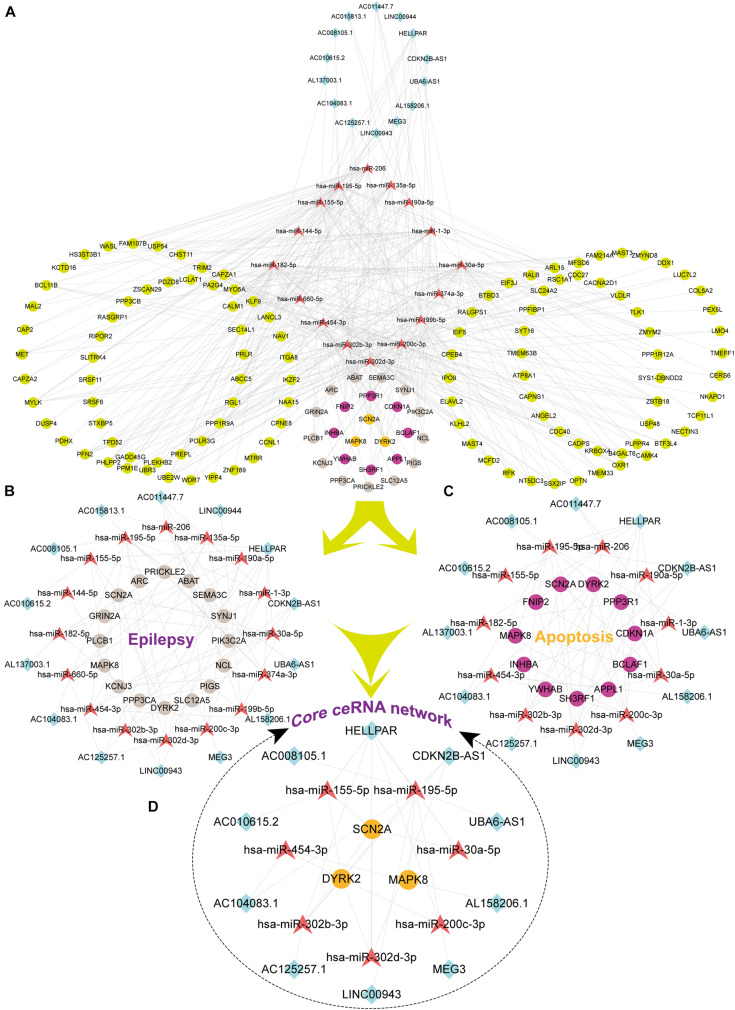
The Down lncRNA-miRNA-mRNA ceRNA network and three sub-networks. Squares represent DElncRNAs, whereas triangles represent DEmiRNAs, and circles represent DEmRNAs. Fuchsia represents up-regulated and other colors represent down-regulated. Light earth color represents genes enriched by Epilepsy in DisGeNet, whereas rose-red represents genes enriched in the apoptotic pathway, and orange color indicates that the genes were enriched both in epilepsy and pathways of apoptotic. **(A)** The Down ceRNA network; **(B)** the ceRNA network of Epilepsy; **(C)** the ceRNA network of Apoptosis; **(D)** the core ceRNA network of Epilepsy and Apoptosis.

We firstly predicted interactions between DElncRNAs and DEmiRNAs via analyzing previously validated CLIP-Seq data sets from the starBase database. A total of 766 lncRNA-miRNA pairs contained 23 DElncRNAs and 29 DEmiRNAs. To further explore the role of DEmiRNAs, we predicted the potential target sites of DEmiRNAs through starBase database. Among the 42 candidate DEmiRNAs, we only detected 22 DEmiRNAs interacting with 222 DEmRNA with 626 predicted pairs, and the remaining 20 DEmiRNAs in the absence of specific DEmRNA targets were left out for further analysis. Ultimately, based on the integration of 766 DEcircRNA-DEmiRNA pairs and 626 DEmiRNA-DEmRNA pair, 29 DElncRNAs, 21 DEmiRNAs, and 221 DEmRNAs were incorporated into the ceRNA regulatory network. According to ceRNA theory and the expression levels of DEmRNAs, we constructed two ceRNA sub-networks with over- or under-expressed DEmRNAs. Specifically, the most enriched up-regulated pathways were organized into the correlation network composed of 5 over-expressed DElncRNAs, 4 under-expressed DEmiRNAs, and 32 over-expressed DEmRNAs, and was visualized in Figure 4. Meantime, The down-regulated network consisted of 14 under-expressed DElncRNAs, 16 over-expressed DEmiRNAs, and 135 under-expressed DEmRNAs as visualized in Figure 5A.

### Functional Enrichment Analysis of up- and Down-Regulated DEmRNAs in the ceRNA Networks

**FIGURE 6:**
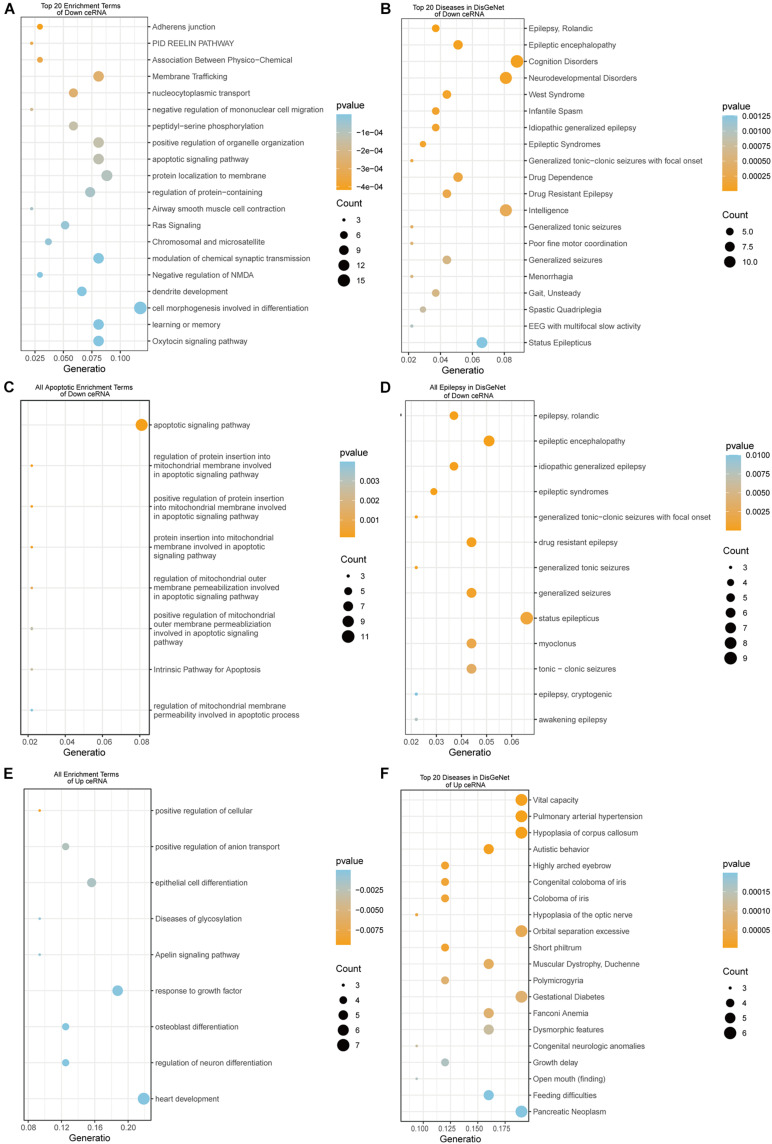
The enrichment results of DEmRNAs in Down and Up ceRNA network. Down ceRNA network enrichment results **(A–D)**. **(A)** Top 20 enrichment results of GO, KEGG, and Reactome; **(B)** top 20 Diseases in DisGeNet; **(C)** all terms related to Apoptosis; **(D)** all types of epilepsy in DisGeNet. UP ceRNA network enrichment results **(E,F)**. **(E)** Top 20 enrichment results of GO, KEGG, and Reactome; **(F)** top 20 diseases in DisGeNet.

**FIGURE 7:**
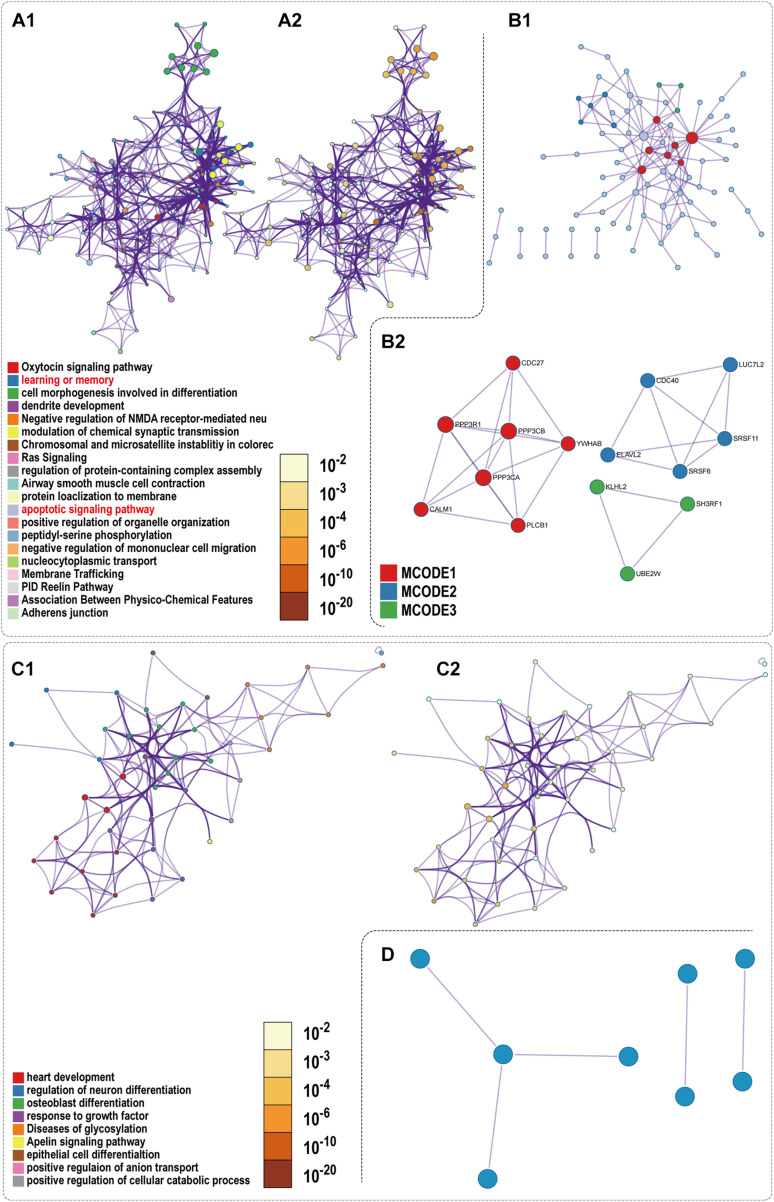
PPI network and enrichment terms of mRNAs of Down ceRNA and Up ceRNA. **(A1,C1)** Colored by cluster ID; **(A2,C2)** colored by *p*-value; **(B1,D)** Protein-protein interaction network; **(B2)** MCODE components.

**FIGURE 8:**
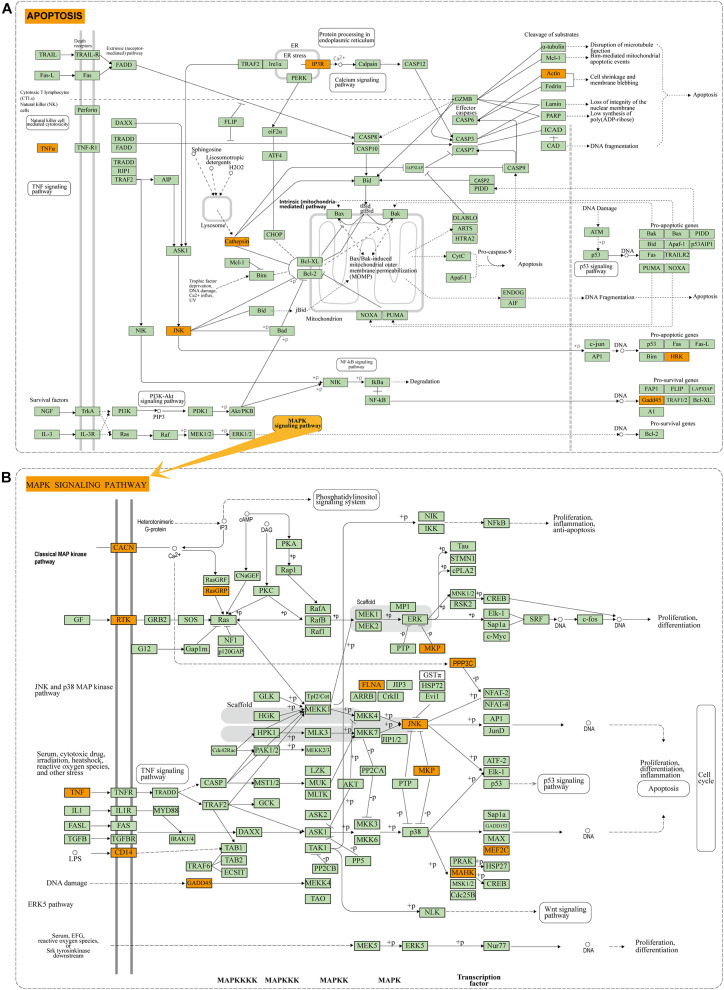
Enrichment results of KEGG pathway: **(A)** Apoptosis pathway of DEmRNAs in down-regulated ceRNA network. **(B)** MAPK pathway of DEmRNAs in down-regulated ceRNA network. Orange module indicates the enriched DEmRNAs in the apoptosis and MAPK pathway.

To reveal the biological function of the lncRNA mediated ceRNA network, we conducted functional enrichment analyses of these up- and down-regulated DEmRNAs in the ceRNA networks based on GO terms and KEGG pathways annotations utilizing Metascape program. The top 20 enriched GO functions for downregulated DEmRNAs were mainly involved in adherens junction, neuronal apoptosis, apoptotic signaling pathway, modulation of chemical synaptic transmission, negative modulation of NMDA receptors, dendrite development, regulation of neuron differentiation and cognitive functions (Figures 6A, 7A1,A2). Similarly, enrichment results from DisGeNET database were also dominated by the pathways of epileptic seizure or other neurological disorders (Figure 6B). Overall, 8 GO terms in the analysis were related to apoptosis and 13 entries were associated with epileptogenesis in the down-regulated ceRNA networks (Figures 6C,D). In order to investigate the key genes in HS-TLE, we identified two main pathways including MAPK signaling pathway, apoptosis pathway and relationships among DEmRNAs by pathway-act-network (Figure 8), which might represent potential therapeutic targets for TLE. To recognize the interacting pattern in down-regulated DEmRNA, the PPT networks were constructed with 134 nodes and 102 edges (Figure 7B1), which consisted of 3 major functional MCODEs (Figure 7B2). Additionally, the enriched GO functions for up-regulated DEmRNAs were primarily related to regulation of neuron differentiation, response to growth factor, glycosylation, apelin signaling pathway, epithelial cell differentiation, positive regulation of anion transport, and positive regulation of cellular catabolic process (Figures 6E, 7C1,C2). In the DisGeNET database, no up-regulated DEmRNAs were directly linked to epilepsy, while hypoplasia of corpus callosum and congenital neurologic anomalies might be relevant to the genetic epileptic seizures (Figure 6F). The cluster of PPI network (Figures 7C1,C2) and functional MCODEs (Figure 7D) were also constructed for up-regulated DEmRNAs.

### Construction of Neuronal Apoptosis Associated Core ceRNA Network in HS-TLE

Based on the results of functional enrichment analysis, we further divided the down-regulated ceRNA network into two groups: epilepsy-related sub-network (Figure 5B) and apoptosis-related sub-network (Figure 5C). Then by integrating shared connections between two sub-networks, we reconstructed the core ceRNA network concurrently related to epilepsy and neuronal apoptosis (Figure 5D) which consisted of 10 DElncRNAs, 7 DEmiRNAs, and 3 DEmRNAs. Here, we mainly addressed two typical ceRNA cross-talks. The expression level of CDKN2B-AS1 was significantly correlated with 3 up-regulated miRNAs (hsa-miR-195-5p, hsa-miR-302b-ap, and hsa-miR-302d-3p), which in turn led to derepression of its target genes DYRK2, MAPK8, and SCN2A. Alternatively, MEG3 and UBA6-AS1 might act as competitive “sponges” for hsa-miR-195-5p and rescue the expression of DYRK2 and MAPK8. All members of the ceRNA regulatory networks could be seen in the Supplementary Table 2. Validation of key differentially expressed genes in ceRNA network was utilized qRT-PCR.

**FIGURE 9:**
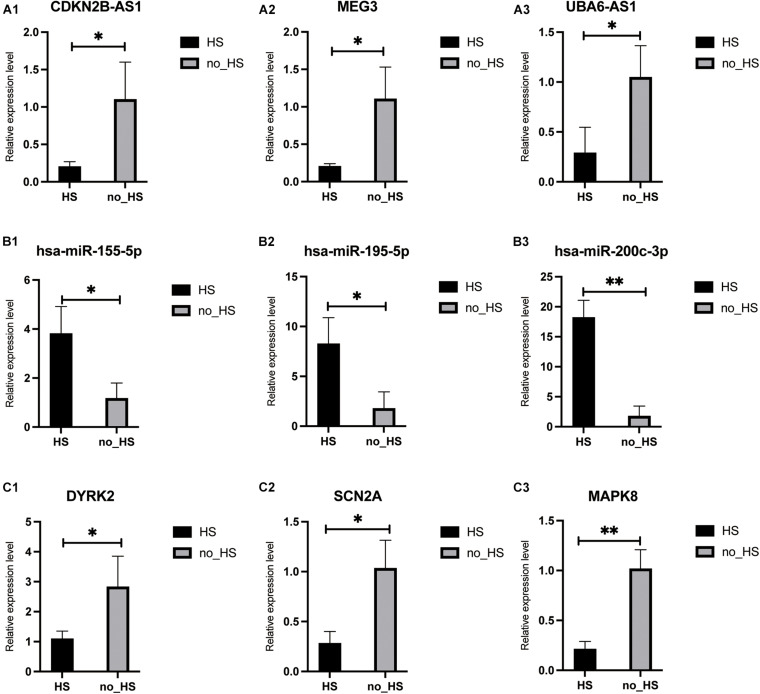
Validation of RNAs in core neuronal apoptosis ceRNA in HS and no-HS patients. All 9 RNA transcripts were verified in a cohort of HS (*n* = 6), no-HS (*n* = 6) hippocampus samples. lncRNA: **(A1–A3)**; miRNA: **(B1–B3)**; mRNA: **(C1–C3)**. ^∗^*p* < 0.05, ^∗∗^*p* < 0.001.

To further verify the results of RNA-seq and small RNA-seq, qRT-PCR was performed to detect the expression levels of nine genes which were chosen based on the absolute value of Log2FoldChange in the neuronal apoptosis ceRNA network. As shown in Figure 9, the results of qRT-PCR confirmed that miRNAs including *hsa-miR-155-5p, hsa-miR-195-5p, and hsa-miR-200c-3p* were significantly up-regulated, while lncRNAs including *CDKN2B-AS1, MEG3, UBA6-AS1*, and protein-coding RNAs including *SCN2A, DYRK2, and MAPK8* were significantly downregulated in the 6 HS samples compared with 6 no-HS samples in accordance with RNA-sequencing analyses.

## Discussion

Epilepsy is caused by abnormal synchronous electrical discharge of neurons, and repeated episodes might accelerate neuronal injury and result in re-organization of local neural network, which in turn irritate seizures and contribute to neurocognitive impairments (Goldberg and Coulter, 2013). Recently, numerous studies have described aberrant expression of protein-coding and non-coding genes with potential roles in epileptogenesis (Noebels, 2015), whereas understanding the complex interaction between them remains a formidable task in human epileptic research. In the present study, we explored the mechanisms underlying epilepsy-induced hippocampal neurons apoptosis in CA1 and CA4 subregions by high throughput RNA-seq of HS-TLE samples and non-HS-TLE samples. A total of 457 DEmRNAs, 381 DElncRNAs, and 42 DEmiRNAs were identified across the transcriptomes in HS group after compared with non-HS group. Taking a step further, we constructed for the first time a neuronal apoptosis associated ceRNA network for TLE through integrative bioinformatic analysis on human samples, which provide novel therapeutic targets for future research. In addition, the GSEA analysis indicted HS-related functional gene sets involved in cognitive impairment, inflammatory response, MAPK and extrinsic apoptotic signaling pathway. Consistent with these findings, it has been reported that uncontrolled neuroinflammatory response modulated by lncRNAs drives the process of epilepsy and exacerbates seizure frequency (Wan and Yang, 2020), which might promote apoptosis of hippocampal neurons (Fang et al., 2019) and impair learning-memory function in HS patients. Notably, several key genes in the ceRNA network were verified by qRT-PCR.

Neuronal cell death in the hippocampus is the key molecular mechanisms underlying HS and cognitive impairment in TLE patients. Based on enrichment analysis of GO and KEGG pathways for targeted genes of DElncRNAs, we revealed MAPK8/JNK and TNF signaling were related to hippocampal neurons apoptosis, while STEAP3 signaling were linked to the regulation of ferroptosis and inflammatory response. Previous studies have illustrated JNK signaling pathway could mediate apoptosis through regulating anti-apoptotic molecules such as Bcl-2 and pro-apoptotic molecules such as Bax (Huang et al., 2015; Duan et al., 2018). Aberrant expression of apoptosis-related genes has been widely reported in TLE tissues and animal epileptic models. For instance, Han et al. observed increased expression of Caspase-3 and decreased expression of Bcl-2 in KA-induced epileptic rats (Han et al., 2018). Consistent with their studies, we also observed downregulation of anti-apoptotic genes (CDKN1A, YWHAB, etc.) and upregulation of pro-apoptotic genes (BCLAF1, etc.) in human epilepsy samples. Collectively, our analysis suggested DElncRNAs might promote caspase-dependent apoptosis through MAPK8/JNK signaling pathway in the course of epilepsy, which have been confirmed by several previous studies in epileptic rat models (Wu and Yi, 2018; Xiao et al., 2018; Luo et al., 2019). Based on the ceRNA theory, 10 DElncRNAs, 7 DEmiRNAs, and 3 DEmRNAs comprised the core of apoptosis associated ceRNA network for TLE. The molecular functions of lncRNAs were gradually uncovered in recent years. For instance, CDKN2B-AS1 was reported to up-regulate the expression of GDNF and inhibit neuronal apoptosis by acting as the molecular sponge for miR-133 (Jiang et al., 2021). In addition, the expression of MEG3 was down-regulated in rats with TLE, and overexpression of MEG3 reduced the release of proinflammatory cytokines (including IL-1β, IL-6, and TNF-α) and inhibited neuronal apoptosis via the PI3K/AKT/mTOR pathway in the hippocampus (Zhang et al., 2020). UBA6-AS1 might regulate cell proliferation and apoptosis by competitively binding to miR-7648 and targeting YTHDC1 (Zheng et al., 2021). In this study, we also identified CDKN2B-AS1, MEG3, and UBA6-AS1 dysregulation in TLE through RNA sequencing data and qPCR and these DElncRNAs were involved in the process of neuronal apoptosis and epileptogenesis by targeting downstream miRNAs or protein-coding genes directly. The potential roles of other DElncRNAs in TLE required further functional annotation and experimental validation.

The downstream regulatory mechanisms of lncRNA are multifaceted and complex. LncRNAs can regulate gene expression positively or negatively depending on the cellular context. In this study, all these dysregulated miRNAs of the ceRNA network were involved in apoptosis directly or indirectly in accordance with earlier research and some of them have been studied only in animal models of epilepsy so far. For instance, miR-302 family have been implicated in hippocampal neurogenesis, and down-regulation of miR-302 may contribute to neuronal loss (Ghasemi-Kasman et al., 2017). Duan et al. reported overexpression of miRNA-155 induced neuronal apoptosis in TLE by inhibiting the PI3K/Akt/mTOR signaling pathway (Duan et al., 2018) Other miRNAs (e.g., miR-454, miR-200c) that underwent expression changes in HS-TLE have also been found in relation to neuronal apoptosis (Wang et al., 2020; Zhao et al., 2020; Hu et al., 2021). In this work, we analyzed the potential target genes of these DEmiRNAs, including *DYRK2, SCN2A*, and *MAPK8*, which were significantly down-regulated in HS-TLE group compared with the control group. SCN2A encodes the alpha subunit of the sodium channel neuronal type 2 that mediates normal discharge of neurons and is essential to maintain neuronal homeostasis. Pathogenic variants or dysregulation in SCN2A were associated with a spectrum of related epilepsy syndromes and other neurological disorders (Meisler and Kearney, 2005). MAPK8 participates in a wide range of cellular processes such as proliferation, differentiation, transcriptional regulation and apoptosis (Dhanasekaran and Reddy, 2008). Transient MAPK8 activation could promote cell survival, while prolonged MAPK8 activation might contribute to apoptosis in a context-dependent manner (He et al., 2012). Additionally, Shao et al. reported that MAPK8 signaling pathway might promote drugs resistance in patients with refractory epilepsy via the modulation of P-glycoprotein expression (Shao et al., 2016). In our study, MAPK8 play a pivotal role in neuronal apoptosis regulated by several DElncRNAs in HS-TLE. DYRK2 belongs to the class II DYRK subfamily, which mediates the phosphorylation of p53 at Ser46 to potentiate p53-dependent apoptosis (Taira et al., 2007). We found DYRK2 were desregulated and related to the progression of hippocampal sclerosis in TLE patients.

Undeniably, our study has several limitations. Firstly, owing to the stringent filtering criteria and the relative scarcity of hippocampus tissue, the sample size of each group in this study was rather small. Therefore, future work should seek to replicate these findings in multicenter cohort studies with larger sample sizes. Secondly, the bulk sequencing technique does not allow us to attribute these changes in gene expression to specific cell populations in the hippocampus. We next aim to validate these observations with whole genome single-cell sequencing by using our own surgical samples. Finally, it remains unclear whether differential expressed RNAs in the ceRNA network represent a cause or consequence of HS and hippocampal neurons apoptosis. In this direction, further in depth functional validation in relevant cell or animal models of TLE are warranted to verify our findings, and ongoing investigations of these issues are current under way in our laboratory. Despite the limitations, our study constructed the first neuronal apoptosis associated ceRNA network in human TLE, which displayed a novel ceRNA regulatory mechanism of hippocampal sclerosis in TLE patients for further investigation.

In summary, we identified a LncRNA-mediated ceRNA network consisting of 10 lncRNAs, 7 miRNAs, and 3 mRNAs for neurons apoptosis in HS-TLE patients, and some of these hub genes were firstly reported in epileptogenic process by this study. This study provided a novel insight into the complex regulatory network of differential expressed RNAs that mediated neuronal loss in HS, which need to be further evaluated in functional experiments for their potential as predictive biomarkers and therapeutic targets in HS-TLE.

## Data Availability Statement

The RNA raw sequencing data has been stored in the Sequence Read Archive (SRA) database with the registration number PRJNA699348 and raw sequence data of the micro RNA has been submitted to GEO (GSE124507).

## Ethics Statement

The studies involving human participants were reviewed and approved by The First Clinical College Ethics Committee of Harbin Medical University. The patients/participants provided their written informed consent to participate in this study. Written informed consent was obtained from the individual(s) for the publication of any potentially identifiable images or data included in this article.

## Author Contributions

ZL and MN conceived and designed this study and reviewed the literature for this manuscript before finalizing its submission. HW, LL, and HS collected the sample for this study. JW and LM extracted RNA from the samples and performed RNA-Seq. SYa and AW perform qPCR validation. SYu analyzed the data and wrote the manuscript. YG and TW edited the manuscript (figures and language). All authors read and approved the final manuscript.

## Conflict of Interest

The authors declare that the research was conducted in the absence of any commercial or financial relationships that could be construed as a potential conflict of interest.

## Publisher’s Note

All claims expressed in this article are solely those of the authors and do not necessarily represent those of their affiliated organizations, or those of the publisher, the editors and the reviewers. Any product that may be evaluated in this article, or claim that may be made by its manufacturer, is not guaranteed or endorsed by the publisher.

## Footnotes

1
http://starbase.sysu.edu.cn/

2
http://metascape.org

## Abbreviations

ceRNA: Competing endogenous RNA
TLE: Temporal lobe epilepsy
HS: Hippocampal Sclerosis
lncRNAs: long non-coding RNAs
DEmRNAs: differential expression mRNAs
DElncRNAs: differential expression lncRNAs
DEmiRNAs: differential expression miRNAs
PPI: Protein-Protein interaction
GO: Gene Ontology
KEGG: Kyoto Encyclopedia of Genes and Genomes
GSEA: Gene Set Enrichment Analysis
DEGs: Differential expression genes
ILEA: League Against Epilepsy.
