# Supplementary material for: Study of Neuronal Apoptosis ceRNA Network in Hippocampal Sclerosis of Human Temporal Lobe Epilepsy by RNA-Seq
Source: Front Neurosci. 2021 Nov 12;15:770627. doi: 10.3389/fnins.2021.770627 (PMC8633546; doi:10.3389/fnins.2021.770627)
Supplement: Supplementary file 1 [file Data_Sheet_1.pdf]

Supplementary Appendix

Supplementary Table S1. Clinical data of HS-TLE and nonHS-TLE patients

Group	Patient	Gender	Age at First seizure (year)	Duration of epilepsy(year)	seizure type	seizure frequency (times/month)	Seizure duration (seconds)	Side of Hippocampus	Age at surgery (years)	AEDs	ILAE classification	RNA-Seq
TLE-HS	1	M	9	15	GAS	2~10	60~600	Left	24	PHB LEV VPA	ILAE-I	Yes
	2	M	9	20	GAS	3~4	60~120	Left	29	CBZ LTG		
	3	F	4	25	CTCS	3~9	180~600	Left	29	VPA LEV TPM		
	4	M	7	8	GSA	10~90	5~60	Left	15	VPA OXC LTG LEV		No
	5	F	18	3	GS	irregular	3~600	Right	21	CBZ VPA		
	6	F	8	4	GS	irregular	4~10	Right	12	OXC LEV LTG		
	7	F	13	14	PZ or + GSA	PZ: 8~9; GSA: 2~3	2~600	Left	27	PHB CBZ TPM		
TLE-nonHS	8	M	22	5	GTCS	3~6	240~420	Left	27	PHB VPA TPM	no-HS	Yes
	9	M	29	2	GTCS	1~3	60~480	Right	31	OXC VPA LTG		
	10	F	27	3	GTCS	irregular	3~60	Right	30	PHB LEV VPA		
	11	M	9	10	GAS	4~10	60~300	Right	19	PHB OXC		No
	12	M	14	15	GS	4~7	5~60	Left	29	VPA OXC CBZ		
	13	F	14	10	GTCS	2~5	60~480	Right	24	CBZ OXC LEV		
	14	F	20	9	GTCS	1~7	10~120	Right	29	LEV VPA		

Abbreviations: AEDs: antiepileptic drugs, CBZ: carbamazepine, LEV: levetiracetam, LTG: lamotrigine, PHB: phenobarbital, TPM: topiramate, VPA: valproic acid, OXC: oxcarbazepine. PZ: partial seizure, GS: generalized seizures, GAS: generalized absence *seizure,* GMS: generalized myoclonic seizures, GTCS: generalized tonic clonic seizures, GSA: generalized seizures with atonic.

Supplementary Table S2. Gene lists in the ceRNA network

mRNA	lncRNA	miRNA
**Apoptotic ceRNA network**		
APPL1	AC104083.1, AL137003.1, HELLPAR	has-miR-454-3p, has-miR-182-5p, has-miR-200c-3p
BCLAF1	AL137003.1, HELLPAR	has-miR-182-5p, has-miR-30a-5p
CDKN1A	AC104083.1	has-miR-454-3p
DYRK2	AC104083.1, AC125257.1, CDKN2B-AS1, HELLPAR, MEG3, UBA6-AS1	has-miR-195-5p, has-miR-454-3p, has-miR-302b-3p, has-miR-302d-3p, has-miR-155-5p, has-miR-200c-3p
FNIP2	AC011447.7, AC104083.1, AC125257.1, CDKN2B-AS1, HELLPAR, MEG3, UBA6-AS1	has-miR-206, has-miR-195-5p, has-miR-302b-3p, has-miR-302d-3p, has-miR-30a-5p
INHBA	HELLPAR	has-miR-200c-3p
MAPK8	AC104083.1, CDKN2B-AS1, HELLPAR, MEG3, UBA6-AS1	has-miR-195-5p
PPP3R1	AC125257.1, AL137003.1, CDKN2B-AS1, HELLPAR	has-miR-302b-3p, has-miR-302d-3p, has-miR-182-5p, has-miR-200c-3p, has-miR-30a-5p
SCN2A	AC125257.1, CDKN2B-AS1, HELLPAR	has-miR-302b-3p, has-miR-302d-3p, has-miR-155-5p, has-miR-30a-5p
SH3RF1	AC011447.7, HELLPAR	has-miR-1-3p, has-miR-206, has-miR-30a-5p
YWHAB	AC104083.1, HELLPAR	has-miR-454-3p, has-miR-200c-3p
**Epilepsy ceRNA network**		
ABAT	AC015813.1, AC125257.1, HELLPAR	hsa-miR-135a-5p, hsa-miR-660-5p, hsa-miR-155-5p, hsa-miR-200c-3p
ARC	AC015813.1, AC125257.1	hsa-miR-135a-5p
DYRK2	AC104083.1, AC125257.1, CDKN2B-AS1, HELLPAR, MEG3, UBA6-AS1	hsa-miR-195-5p, hsa-miR-454-3p, hsa-miR-302b-3p, hsa-miR-302d-3p, hsa-miR-155-5p, hsa-miR-200c-3p
GRIN2A	HELLPAR	hsa-miR-30a-5p
KCNJ3	AC011447.7, AC015813.1, HELLPAR, UBA6-AS1	hsa-miR-374a-3p, hsa-miR-30a-5p
MAPK8	AC104083.1, CDKN2B-AS1, HELLPAR, MEG3, UBA6-AS1	hsa-miR-195-5p
NCL	AC011447.7	hsa-miR-1-3p, hsa-miR-206
PIGS	AC011447.7, AC104083.1, CDKN2B-AS1, LINC00944	hsa-miR-199b-5p, hsa-miR-454-3p
PIK3C2A	AC011447.7, AC015813.1, AC104083.1, AC125257.1	hsa-miR-1-3p, hsa-miR-206, hsa-miR-135a-5p, hsa-miR-454-3p
PLCB1	AC015813.1, AC104083.1, AC125257.1, HELLPAR	hsa-miR-135a-5p, hsa-miR-454-3p, hsa-miR-155-5p
PPP3CA	AL137003.1, HELLPAR	hsa-miR-182-5p, hsa-miR-30a-5p
PRICKLE2	AC011447.7, AC015813.1, AC104083.1, AL137003.1, HELLPAR, UBA6-AS1	hsa-miR-374a-3p, hsa-miR-454-3p, hsa-miR-182-5p
SCN2A	AC125257.1, CDKN2B-AS1, HELLPAR	hsa-miR-302b-3p, hsa-miR-302d-3p, hsa-miR-155-5p, hsa-miR-30a-5p
SEMA3C	AC125257.1, CDKN2B-AS1, HELLPAR	hsa-miR-302b-3p, hsa-miR-302d-3p
SLC12A5	AC104083.1	hsa-miR-454-3p
SYNJ1	AC011447.7, AC104083.1, CDKN2B-AS1, HELLPAR, MEG3, UBA6-AS1	hsa-miR-1-3p, hsa-miR-206, hsa-miR-195-5p, hsa-miR-454-3p, hsa-miR-200c-3p
**Core neuronal apoptosis ceRNA network**		
DYRK2	AC104083.1, AC125257.1, CDKN2B-AS1, HELLPAR, MEG3, UBA6-AS1	has-miR-195-5p, has-miR-454-3p, has-miR-302b-3p, has-miR-302d-3p, has-miR-155-5p, has-miR-200c-3p
MAPK8	AC104083.1, CDKN2B-AS1, HELLPAR, MEG3, UBA6-AS1	has-miR-195-5p
SCN2A	AC125257.1, CDKN2B-AS1, HELLPAR	has-miR-302b-3p, has-miR-302d-3p, has-miR-155-5p, has-miR-30a-5p

Supplementary Table S3. Primers of qRT-PCR

Type	Gene	Strand	Sequence
miRNA	hsa-miR-195-5p	F	TAGCAGCACAGAAACAT
	hsa-miR-155-5p	F	AATGCTAATCGTGATAGGGGT
	hsa-miR-200c-3p	F	TAATACTGCCTGGTAATGATG
mRNA	DYRK2	F	GCTCCACCACCAAAGATCCC
		R	GCACCTAAAGGAGAGGGCTG
	MAPK8	F	CGCCGTTCGTCAGCTTCA
		R	TACTGCCGCCAATCGTGT
	SCN2A	F	TCTTCTTGGTGCCAGCTTATCA
		R	GTGCCATCTTTTCATCCTGCT
lncRNA	CDKN2B-AS1	F	TCTGACGCGACATCTGGAC
		R	AAAAGGGACACTAGTCCGGC
	MEG3	F	GCTTAGTTCCTGGCACTCTGA
		R	AGCTCATTAGCACGGTAACCA
	UBA6-AS1	F	TGCTCTACAGCCAAACATGC
		R	CTGAAAGGCTTTGGGACTTTGA
Inner Reference			
mRNA<br>lncRNA	GAPDH	F	GTCTCCTCTGACTTCAACAGCG
		R	ACCACCCTGTTGCTGTAGCCAA
miRNA	RNU6b	F	TTCGTGAAGCGTTCCATATTTTTTCGTGAAGCGTTCCATATTTT

Supplementary Figure S1. Immunohistochemical NeuN staining of hippocampal neurons

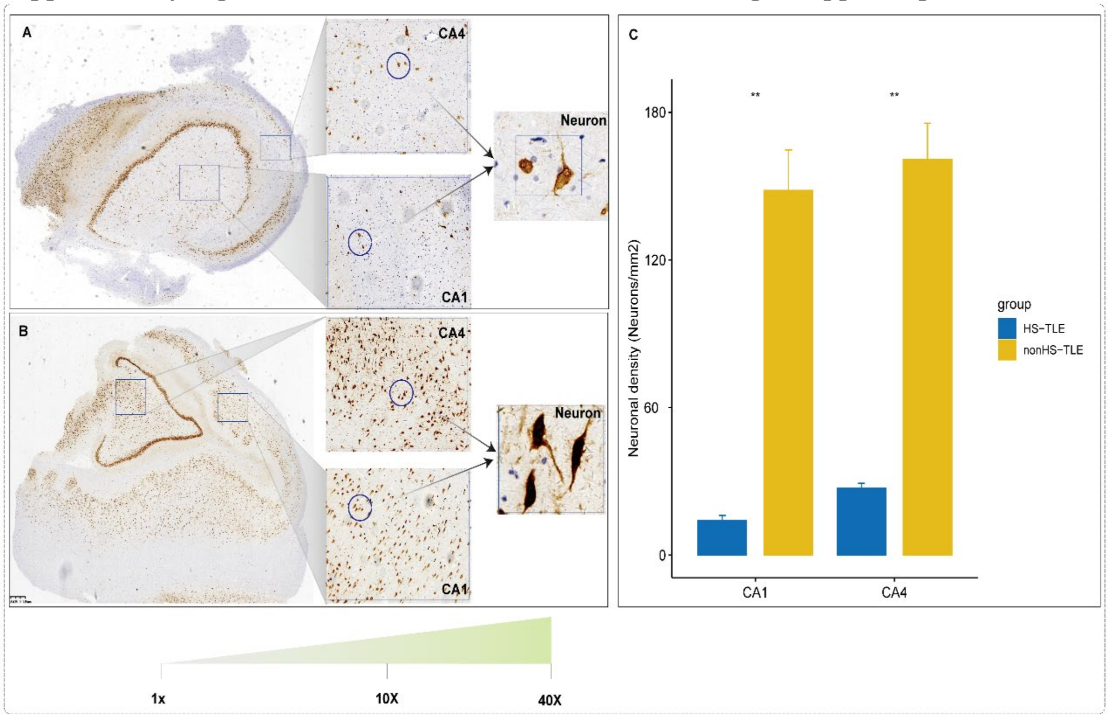

Immunohistochemical NeuN staining of HS-TLE and nonHS-TLE group. A. Low-magnification view (×1) of the HS-TLE group showing the neuronal loss in the CA4 and CA1 regions; High-magnification view (×10) of the HS-TLE group in CA4 and CA1; B. Low-magnification view (×1) of the nonHS-TLE group showing no neuronal loss in the CA4 and CA1; High-magnification view (×10) of the no-HS group in CA4 and CA1. C. Comparison of neuronal density between the HS-TLE and nonHS-TLE groups（** $p \leq 0.01$）.
